# Terminology of e-Oral Health: Consensus Report of the IADR’s e-Oral Health Network Terminology Task Force

**DOI:** 10.1186/s12903-024-03929-z

**Published:** 2024-02-28

**Authors:** Rodrigo J. Mariño, Sergio E. Uribe, Rebecca Chen, Falk Schwendicke, Nicolas Giraudeau, Janneke F. M. Scheerman

**Affiliations:** 1 Center for Research in Epidemiology, Economics and Oral Public Health (CIEESPO), Faculty of Dentistry, Universidad de La Frontera, Temuco, Chile; 2 Melbourne Dental School, University of Melbourne, Melbourne, Australia; 3 Faculty of Dentistry, University of Valparaiso, Valparaiso, Chile; 4 Department of Conservative Dentistry and Oral Health, Riga Stradins University, Riga, Latvia; 5 Baltic Biomaterials Centre of Excellence, Headquarters, Riga Technical University, Riga, Latvia; 6 Westmead Applied Research Centre, Faculty of Medicine and Health, The University of Sydney, Sydney, New South Wales Australia; 7 Clinic for Conservative Dentistry and Periodontology, LMU University Hospital, LMU, Munich, Germany; 8 Division CEPEL Organization CNRS, University of Montpellier, 163 rue Auguste Broussonnet, Montpellier, 34090 France; 9 Department of Oral Healthcare; Health, Sports and Welfare, InHolland University of Applied Sciences, Gustav Mahlerlaan 3004, Amsterdam, Noord-Holland 1081LA The Netherlands

**Keywords:** Terminology, Teledentistry, E-Oral health, Consensus, Dentistry, Digital health

## Abstract

**Objective:**

Authors reported multiple definitions of e-oral health and related terms, and used several definitions interchangeably, like mhealth, teledentistry, teleoral medicine and telehealth. The International Association of Dental Research e-Oral Health Network (e-OHN) aimed to establish a consensus on terminology related to digital technologies used in oral healthcare.

**Method:**

The Crowdsourcing Delphi method used in this study comprised of four main stages. In the first stage, the task force created a list of terms and definitions around digital health technologies based on the literature and established a panel of experts. Inclusion criteria for the panellists were: to be actively involved in either research and/or working in e-oral health fields; and willing to participate in the consensus process. In the second stage, an email-based consultation was organized with the panel of experts to confirm an initial set of terms. In the third stage, consisted of: a) an online meeting where the list of terms was presented and refined; and b) a presentation at the 2022-IADR annual meeting. The fourth stage consisted of two rounds of feedback to solicit experts’ opinion about the terminology and group discussion to reach consensus. A Delphi-questionnaire was sent online to all experts to independently assess a) the appropriateness of the terms, and b) the accompanying definitions, and vote on whether they agreed with them. In a second round, each expert received an individualised questionnaire, which presented the expert’s own responses from the first round and the panellists’ overall response (% agreement/disagreement) to each term. It was decided that 70% or higher agreement among experts on the terms and definitions would represent consensus.

**Results:**

The study led to the identification of an initial set of 43 terms. The list of initial terms was refined to a core set of 37 terms. Initially, 34 experts took part in the consensus process about terms and definitions. From them, 27 experts completed the first rounds of consultations, and 15 the final round of consultations. All terms and definitions were confirmed via online voting (i.e., achieving above the agreed 70% threshold), which indicate their agreed recommendation for use in e-oral health research, dental public health, and clinical practice.

**Conclusion:**

This is the first study in oral health organised to achieve consensus in e-oral health terminology. This terminology is presented as a resource for interested parties. These terms were also conceptualised to suit with the new healthcare ecosystem and the place of e-oral health within it. The universal use of this terminology to label interventions in future research will increase the homogeneity of future studies including systematic reviews.

## Introduction

The use of digital technologies in healthcare (digital health) has been transformative. Digital health can improve access, provide continuity, improve efficiency in delivery and increase the quality of healthcare [1]. The uptake of digital technologies has grown considerably over the last decade, with the increased presence of the internet and mobile technologies [2]. In rural and hard to access areas in particular, alongside the unprecedented globality of mobile connectivity, digital health presents as a transformative agent that removes geographical barriers to healthcare [3].

During the COVID-19 pandemic, health professionals, almost overnight, needed to find ways to provide alternatives to face to face consultation and hands-on therapies [4, 5]. Consequently, health organisations and providers experienced years of digital transformation in the space of months.

In oral healthcare, the use of digital technologies has also accelerated in recent years [6]. The World Health Organization (WHO) encourages the implementation of digital health and digital solutions for oral health [7]. The Guiding principles for Global Strategy on Oral Health include “Optimising digital technology for oral health” [8]. WHO has been developing plans to accelerate the use of digital technologies to meet global public health needs based on the World Health Assembly Digital Health resolution (*WHA71*.*7*) [9]. Furthermore, the World Health Assembly Resolution on Oral Health (WHA74.5) [10] outlines broad actionable frameworks and plans that specifically include provisions for the use of modern digital technology in the fields of telemedicine and teledentistry to ensure no one is left behind.

Digital health has many stakeholders, including clinicians, patients, governmental decision-makers, health technologists, researchers and their funders in industry and the public arena. As a result of this multitude of actors and their varied backgrounds, coupled with the rapid rise of digital health and its diversification, there is a high variety of terms and concepts related to the use of technology within health care [11]. The intersection of health, medicine, dentistry, and technology involves a range of digital health terms, concepts and services that are used imprecisely and inconsistently, causing confusion. Terms such as eHealth, mobile Health, telemedicine, artificial intelligence, and health informatics are defined and understood differently by a diverse range of stakeholders. Even the term teledentistry has different meanings, partly caused by the different uses and translations of the term ‘dentistry’ across countries.

WHO has recognized that a shared and standardized vocabulary is necessary to identify knowledge gaps and avoid unnecessary research duplication [11]. Standardisation of digital health terminology is also critical to ensuring that the evaluation of the implementation and effectiveness of digital health interventions is performed using common language. This fosters collaboration and a common understanding between stakeholders, supporting the continued implementation of effective digital health programs [12]. From this perspective, and to provide a common language, and a better understanding of digital terminology used in oral health, the International Association of Dental Research (IADR) e-Oral Health Network (eOHN) (https://www.e-oralhealth.org) board discussed the need to arrive at a common understanding of the terms used in digital technologies in oral healthcare.

The overall aim of this study was the establishment of a consensus terms related to digital technologies in oral healthcare to provide a common, or a bridging, language for the oral health community to articulate current use and progress in digital and mobile health technologies. This paper describes the consensus development process and presents the outcome of the discussions, in terms of a list of terms and definitions as a resource to suit with a new healthcare ecosystem and the place of e-oral health within it [13] and propose a model and methodology for future updates of terms and definitions in digital oral health.

## Methods

### Design

**Fig. 1:**
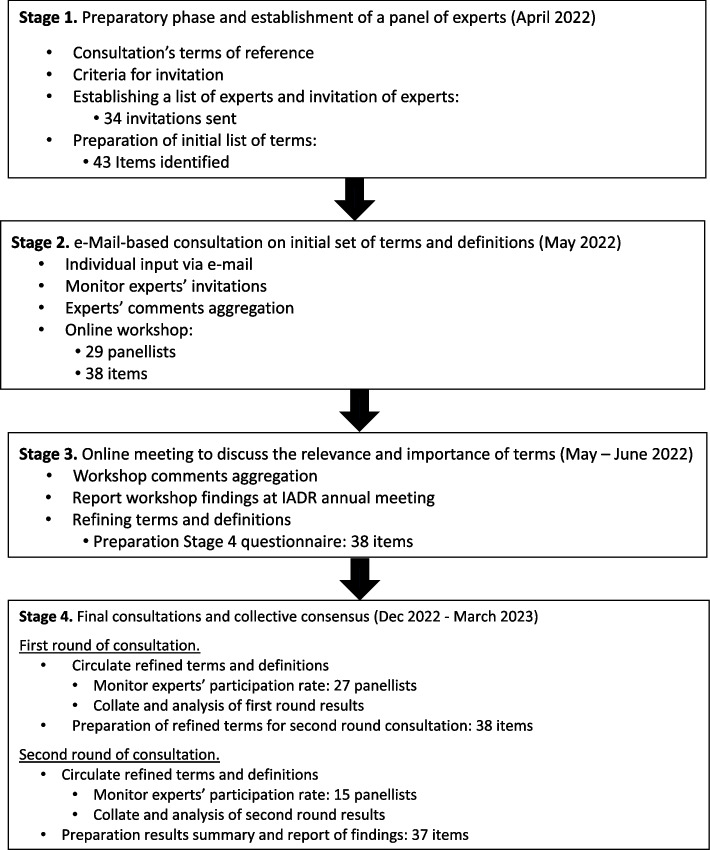
Framework, flowchart, and results of the consensus process

The consensus development followed conventional approaches on consensus building and recommendation from international crowdsourcing/Delphi [14, 15]. The consensus building process comprised four main stages (see Fig. 1):

1. Preparatory phase and establishment of a panel of experts.
2. e-Mail-based consultation on the initial set of terms and definitions.
3. Online meeting to discuss the relevance and importance of terms.
4. Final consultations and collective consensus.

The consensus process was monitored by the task force, which consisted of board members of the eOHN (RM, JS, RC, SU, NG).

Ethics approvals for the study protocol was received from Research Ethics Committee, Riga Stradins University, Riga, Latvia (Nr. 2-PĒK-4/236/202). All participants provided informed consent to take part in the consensus process and online questionnaires.

Stage 1) Preparatory phase and establishment of a panel of experts

It was agreed that the task force would map out and scope the structure of the glossary around the use of digital health technology and solutions in oral health. An initial selection of terms and definitions took place in April 2022. The selection was established based on consensus among the task force members and informed by the literature. To increase the extent of the glossary, a range of documents, reports and publications were reviewed for relevant terms. Definitions were collected from documents issued by WHO [16–20], the Commission of the European Communities on e-Health [21–24], as well as from peer-reviewed journals [25–39], and other relevant resources from professional organisations [40–45]. These definitions were adapted from the literature, while others were a synthesis of different definitions. All terms and definitions were uploaded onto a document where invited experts could leave comments and suggestions.

The task force identified and convened experts in the field of e-oral health to participate in an online consultation process. This panel of experts was identified from two sources: via membership of the IADR’s-eOHN network; and via the task force’s personal networks. Sampling was purposive to ensure that invited experts met the following inclusion criteria: i) actively involved in either research and/or working in e-oral health fields; and ii) willing to participate in the consensus process.

Stage 2). e-Mail-based consultation on the initial set of terms and definitions

To reach a consensus, the task force used Crowdsourcing Delphi [14]. This is the process of aggregating crowd wisdom to solve a problem [14, 46]. Such an approach has been shown to offer more accurate decisions than the sum of individual judgements [15, 47]. This method consists of rounds of feedback and group discussion for aggregated consensus. Thus, email-based consultations and online meetings were organized with the panel of experts. This stage included an e-mail based asynchronous activity with the panel of experts establish a shortlist of terms and definitions to be included in this consensus. The experts were invited to review the document online and asked to suggest further terms that could be added (or deleted) to this consensus document and to refine the definitions regarding teledentistry and digital health.

This stage was broad and iterative with the goal of identifying additional terms and definitions, and reviewing, sending comments, and further refining existing definitions. It was made clear to contributors that this was a brainstorm on potential terms to be included, and all initial terms would be considered. The consultation was decentralised, open for a six-week period, and allowed for independence of judgements where the panel of experts provided feedback in their own time [47]. The resultant document of aggregated terms and definitions was used by the task force members as a basis for subsequent discussions.

Stage 3) Online meeting to discuss the relevance and importance of terms 

After this six-week period, all agreed definitions were revisited by task force members. The refined draft document was circulated within the panel of experts in preparation for the online discussion. The first and last author (JS and RM) organized an online meeting on the 31st of May 2022 with all invited participants to discuss the contents of the circulated draft document and to work towards consensus on the terms and definitions.

The document with initial results from experts consultation would be the matter of a symposium presentation at the 2022 IADR annual meeting.

Stage 4) Final consultations and collective consensus

A fourth stage consisted of two rounds of consultations to determine collective consensus. Via an online Delphi-questionnaire, the panel of experts were first were solicited to independently assess: a) the appropriateness of the terms, and b) the accompanying definitions, and vote on whether or not they agreed with them. That is, whether the definition was suitable to define the term and the conceptual models explained the content well, or not. For each definition, experts were given the option of selecting ‘I do not know’ as an alternative response. A free-text box was available for each term to provide opportunities to explain and/or expand responses.

The task force discussed the results from the first round. Each response was considered a vote and it was assumed that the most accurate definition would get more votes [48]. Majority voting is the most common and simple consensus-based method in crowdsourcing [48]. It was decided that 70% or higher agreement among experts on the terms and definitions would represent consensus [49]. All ‘I do not know’ responses were excluded from the panellists’ overall response. Two reminders were sent to the panel during this period.

In a second round, each expert received an individualised questionnaire, which presented the expert’s own responses from the first round and the panellists’ overall response (% agreement/disagreement) to each term. The experts were asked to reconsider their responses, considering the panellists’ overall response and to assess the clarity of the conceptual models. The results of this round were discussed and analysed by the members of the task force.

## Results

An initial set of terms and definitions identified 43 terms which were circulated within the panel of experts during April – May 2022. Panel of experts were invited to participate and e-mail-based consultation. Thirty-four experts accepted the invitation and reviewed terms organised by the task force to identify gaps, avoid duplications, and suggested new ones.

The eOHN task force met online to discuss the document containing the terms reviewed and/or suggested by panel participants. This document was used to guide the group discussion held on the 31st of May, when 29 participants took part, JS and RM acted as facilitators. The online group meeting lasted 2 hours and the discussion focused on organizing the terms and definitions. This panel included expert from 14 countries representing six continents (i.e., Africa; Asia; Australia; Europe; South and North America). The majority of them (90.5%) with an oral health professional background.

In this meeting, the relevance and importance of terms were discussed to further refine the glossary. Although this initial list provided a workable framework, and there was consensus on several terms, most of the discussion highlighted the lack of clarity between different terms varied definitions members had for some of the terms. In particular, the definition of telehealth and teledentistry varied within jurisdictions. Many jurisdictions also had varied interpretations of the scope of teledentistry and telemedicine.

**Fig. 2:**
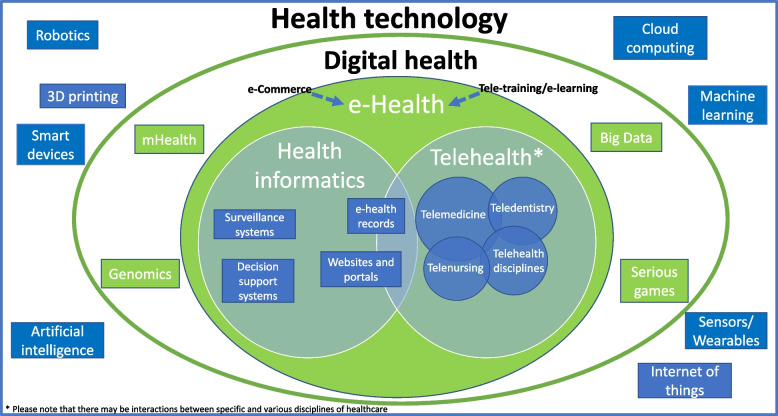
Taxonomy of major components of health technology, digital health and e-health and technologies that support them

Given the heterogeneous terminology used to conceptualise digital oral health, this online workshop assisted taskforce members to develop and build on existing conceptual models used to differentiate the interrelated terms (e.g., health technology, digital health, and e-health), which are often used interchangeably and as synonyms. To clarify the taxonomy of digital health terms, Fig. 2 illustrates the hierarchical relationships among them.

To further conceptualise and organize e-health terms, Fig. 2 follows a recommendation by Richard Scott [50] who proposed that the main components of e-health are telehealth and health Informatics. Accordingly**,** e-health encompasses elements from information systems (i.e., health informatics), to facilitate health acts performed at a distance (i.e., telehealth). Telehealth is the umbrella term and can exist independently of telemedicine. It differs from the specificity of telemedicine, telenursing, or teledentistry in that they refer more specifically to remote clinical services within the scope of those professions [51]. However, telemedicine has been intentionally portrayed as bigger as it may have more influence, than other health disciplines.

Figure 2 illustrates some complementary technology that supports e-health, for example, e-learning (the training – awareness, teaching, instruction, and education – side) and e-commerce (the business side) [52], which, by themselves were not considered e-health.

**Fig. 3:**
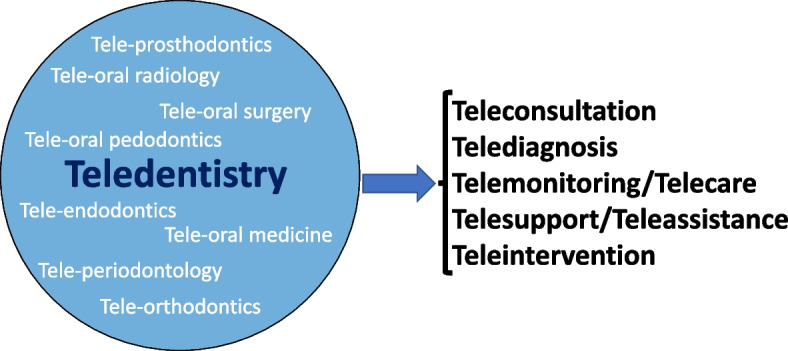
Most common fields of practice in teledentistry and modalities of application

Figure 3 represents how Teledentistry has incorporated the prefix “tele” to common dental clinical disciplines. These terms have evolved to describe the application of teledentistry to those dental specialties, such as “teleprosthodontics”, “teleperiodontology” “teleorthodontics”, “Teleoral surgery” “telepaedodontics”, etc. In contrast to a ‘teledental’ disciplines, the term ‘Teledentistry’ has a more general dental practice meaning [53].

Whilst these models help to conceptualise definitions, the focus of this paper was to outline widely used terms and accepted definitions established during the meetings and discussions. Thus, after the online discussion, from the list of candidate terms, a total of 38 terms were selected for group consultation. These terms and definitions were grouped in alphabetic order. An online survey was open for a 4-week period. Thirty-four invitations to participate in this round of online consultations. From them, 27 experts took part, achieving a response rate of 79.4%.

All 38 terms were agreed on for inclusion. Three terms received full agreement, while six were below 80%, but above the 70% threshold. Major discrepancies in opinions were discussed during an online meeting with the task force members. The free-text responses helped to revise the terms and its accompanying definitions, which were included as statements in the second round. Seven definitions received full agreement and three received agreements between 70 and 80%. The results of this round of consultation were presented by one of the task force members (RM) at IADR’s annual meeting, specifically at the e-oral health network’s symposium on 24th of June 2022 [54].

In view of the task force’s objectives, and in response to the experts’ comments from previous stages of this consultation, further refinements, and additions to the wording of some of the definitions were made. The final list circulated within the panel of experts who replied in the first round (*n* = 27), for a second round of consultations. Fifteen experts replied in this round achieving a response rate of 51.9%. Ten definitions received full agreement, 26 were between 99.0 and 80.1% agreement, and one was below 80%, but above the 70% threshold (see Table 1). One term (i.e., teleorientation) received an agreement below 55% and removed from the list. Table 1 includes the final glossary of 37 terms and definitions for a variety of telehealth and digital oral health technology applications available.

**Table 1:** A glossary of terms used in oral health

Term	Agreement with term to be include in the glossary (%)	Definition	Agreement with term definition (%)
**1. Artificial intelligence in oral health (AI)**	96.6	AI is a field that deals with theory and development of computer systems with an ability to perform tasks that mimic human’s intelligence processes, like problem-solving and decision-making. In oral health AI can support oral health specialists (and connected domains) in early identification of oral diseases, decision making, rapid and reliable data interpretation, workflow automation, treatment monitoring, disease and treatment outcomes prediction and overall improved care quality and patient experience [25].	100.0
**2. Convolutional neural network (CNN)**	79.3	CNNs are a class of artificial neural networks in deep learning. It is a conceptual framework for developing AI algorithms. CNNs are currently used in image and speech analysis, for example [44].	92.9
**3. Deep learning**	86.2	Deep learning is a particularly complex type of machine learning that uses volumes of data and complex algorithms to train a model [26].	85.7
**4. Digital health**	100.0	Digital health is an Umbrella term that comprises e-health (including mobile health) and the use of computational sciences in artificial intelligence, big data and genomics [1]. Digital health extends the concept of e-health to include digital consumers, with a wider range of smart devices and connected equipment. It also encompasses digital health technologies such as the Internet of Things, artificial intelligence, robotics and data analytics [16].	92.9
**5. e-consent**	86.2	A digital or electronic record of a healthcare consumer’s/ patient’s choices, and decision they have voluntarily made to agree to permit more specific treatment, or diagnosis related actions received from health providers [41].	100.0
**6. e-health**	96.6	Is the use of information and communication technology to support health and health-related fields, including healthcare services, surveillance, education, and research [16].	85.7
**7. e-health record**	86.2	A digital repository of patient data that facilitates data entry, interoperability, and transportability of retrospective, current, and prospective information concerning a patient’s health across varied providers and geographic locations, in chronological order [27, 45].	92.9
8. **e-learning**	93.1	e-Learning refers to the use of digital technologies to deliver educational content and support learning in healthcare [28]. e-learning can be provided through a wide range of digital tools and platforms, such as online courses, virtual classrooms, webinars, educational software, and mobile apps.	100.0
**9. e-oral health**	100.0	e-oral health can be described as the use of information and communication technologies in support of oral health care and fields related to oral health care, including oral health surveillance, promotion, education, and research [31].	92.9
**10. e-prescription**	89.7	An electronic time-limited authorization for the provision of medication to the recipient from a licensed authority.	92.9
**11. e-referral**	86.2	The digital exchange of significant patient information from one treating healthcare provider to another via a system of creating, storing and sharing electronic reports [40].	85.7
**12. Gamification**	82.8	Gamification in health is “the application of the characteristics and benefits of games to real-world, non-game processes, problems, or productive activities, and environments, to encourage their users to improve health.” [29, 30].	92.9
**13. Health informatics**	82.8	A discipline or a field of science and engineering that aims at developing methods and technologies for the acquisition, processing, and study of health data, which can come from different sources and modalities, such as electronic health records, diagnostic test results, medical scans [18, 39].	92.9
**14. Health telematics**	82.8	Health-related activities, services, and systemsprovided despite geographical barriers by means of information and telecommunication technologies, for the purposes of global health promotion, disease control and health care, as well as education, management, and research for health [19].	78.6
**15. Digital Health Technology**	82.8	Any digital health technology that aims to enable the management of health systems and services, delivery, and consumption of consumer care, medical care, or broader healthcare [21].	85.7
**16. Health Information and Communication Technologies**	79.3	A set of technologies resulting from the convergence of computing and advanced multimedia and telecommunications techniques, for processing, storage, dissemination, and exchange of health information [16, 42].	85.7
**17. Internet of Things**	79.3	A system of interrelated computing devices or mechanical and digital machines connected to the internet, with the ability to transfer data over a network without requiring human-to-human or human-to computer interaction [16, 31].	85.7
**18. Machine learning in oral health**	96.6	Machine Learning (ML) is a subset of Artificial Intelligence, focused on allowing a machine to automatically learn from data without explicit programming [26].	92.9
**19. m-oral health**	100.0	The use of mobile and wireless technologies (such as mobile phones, tablet computers, and personal digital assistants) to support the achievement of oral health objectives/ dental public health objectives [16, 43].	100.0
**20. Natural language processing**	72.4	An intersection of AI and linguistics that refers to computer systems that analyse, understand, or process human language. Natural language processing (NLP) is a set of automated methods to organize and evaluate the information contained in unstructured clinical notes [32].	92.9
**21. Patient portal for health information**	86.2	A health information technology tool through which patients can access their electronic health records (EHRs) upload additional health information and schedule appointments or interact with providers by chatting to them [33, 34].	85.7
**22. Real-time (synchronous) telehealth consultation**	86.2	Live, two-way interaction between a person (patient, caregiver, or provider) and a health care provider often using audio-visual telecommunications technology [43].	92.9
**23. Robotics / Robotics for healthcare**	82.8	Robotics for healthcare are machines with “systems able to perform coordinated mechatronic actions (force or movement exertions) based on processing information acquired through sensor technology, to support the functioning of impaired individuals, health interventions, care and rehabilitation of patients and also individuals in prevention programs” [22].	85.7
**24. Store-and-forward (asynchronous) telehealth consultation**	89.7	Transmission of recorded health data information (for example, radiographs, photographs, video, digital impressions, and photomicrographs of patients) through an electronic communications system to a health care provider or providers, who uses the information later to evaluate a patient’s condition or render a service outside of a real-time or live interaction [43].	85.7
**25. Teleassistance**	89.7	One health care provider assisting another care provider carrying out specific tasks by means of digital technologies [23].	78.6
**26. Telecare**	82.8	Systems and services where patients and healthcare providers interact remotely to support healthcare delivery by means of digital technologies [23].	92.9
**27. Teleconsultation**	89.7	A consultation made by a healthcare provider with another health care provider or patient via telecommunication technologies; sometimes referred to as remote consultation or virtual consultations, or e-consultations [20].	100.0
**28. Teledentistry**	96.6	Teledentistry represents the uses of Information and telecommunication technology to provide oral healthcare services between an oral healthcare provider and a patient/recipient or other health care providers, who are separated by distance [16, 24].	100.0
**29. Telediagnosis**	86.2	The use of information and telecommunication technology, to establish a diagnosis based on remotely gathered information (e.g., intra-oral cameras) instead of a face-to-face interaction [35].	100.0
**30. Tele-health education**	82.8	A process to promote changes in health attitudes, knowledge, information, behaviours, and skills, by means of information and communication technologies by and for consumers, health professionals and communities, for the purpose of fostering improved health [19]. It may include, audio or video technologies provided synchronously or asynchronously [36].	85.7
**31. Telehealth**	96.6	A collection of information and telecommunications technologies and services that support at-a-distance healthcare delivery and services to a recipient.	92.9
**32. Teleintervention**	72.4	A therapeutic act which is performed remotely by a healthcare provider on a patient/recipient, without or with the local presence of another healthcare provider or providers (e.g., telesurgery) [23].	92.9
**33. Telemedicine**	86.2	The provision of healthcare services using information and telecommunication technology where the health care provider and a patient or another health care provider not in the same location [24].	92.9
**34. Telemonitoring**	89.7	The remote monitoring and evaluation of health and health-related data (e.g., tooth brushing data) between a patient and healthcare provider [37].	100.0
**35. Telesurgery**	79.3	The remote controlling of a surgical apparatus, e.g., a surgical robot, or the remote advice to the surgeon on-site [23].	100.0
**36. Teletriage**	93.1	Evaluation of a patient’s symptoms through remote consultation to establish the need and urgency for face-to-face care [38].	92.9
**37. Wearable Sensors**	86.2	Electronic devices that can be worn on the body to provide real-time sensing information about the wearer, and his/her environment.	100.0

## Discussion

A final set of 37 items and definitions was selected as the result of a consensus building exercise organised to identify common terminology and to arrive at a common understanding of the terms used in digital technologies in oral healthcare. This set of terms was discussed, circulated with several iterations, and agreed on within the task force and the panel of experts. Accordingly, we believe that the terminology list achieved robust consensus.

This is the first attempt in oral health to achieve consensus in e-oral health terminology. These terms are commonly used by health professionals in clinical settings, and by patients using Apps for the self-management of conditions. The glossary of terms also includes terms used in artificial intelligence and machine learning. Their uptake has been rapid across healthcare, and they are becoming a key component in healthcare, sharing expertise, and reducing error and cost [55].

The consensus development followed conventional approaches on consensus building (i.e., a modified Delphi process) [14, 15] and included opinions from around the world. Nevertheless, this attempt to reach consensus was not without limitations. The task force tried as much as possible to fulfill the conditions of crowdsourcing, that is, to allow for diversity of experts, independence of judgments, decentralisation, and aggregation of information [47]. However, it is always challenging to get all the conditions for crowdsourcing and the full implementation was limited by factors such as time and resources. The first concern was related to the self-selection of our expert and obtaining a diverse representation. Our sample was largely composed of experts with an oral health background. Also, although representing six continents, most experts were from a reduced number of countries, which limited the representativeness of the panel. Additionally, the response rate for the second round of consultation of the final Stage was moderate. However, it is possible that this low response rate was due to full agreement with terms in the first round of consultations.

The use of Digital Health technologies (i.e., mobile technology, electronic health records, machine learning, artificial intelligence, etc.) has accelerated the digital transformation of healthcare [56]. This has created a variety of digital health terms, which has led to the need for a standardized vocabulary and consensus on the e-oral health terminology. However, terms and definitions in digital health are dynamic and constantly evolving and changing over time [57]. As digital healthcare progresses, new technologies and terms that both complement and challenge existing ones will arise. Digital technologies will continue to evolve in areas key to oral healthcare improvement. Consequently, although the document may change with the evolving changes in technology, it provides a present state of terms to enable current cohesion and collaborations between those working in digital (oral)health.

## Conclusions

It was considered that the objectives of the International Association of Dental Research e-Oral Health Network (e-OHN) task force were achieved. This Glossary of terms related to digital technologies used in oral health care is presented as a resource that can be used by interested parties. Whilst it provides guidance, and reflects current consensus on terms, it is acknowledged that it will need to remain adaptive to the rapid technological changes in healthcare; this glossary is to be considered a living document that will be updated on a regular basis. Accordingly, a consensus model based on crowdsourcing could be used in future updates of this terminology list was developed.

## Data Availability

The data supporting the findings of this study are available in the Zenodo repository Dataset - Terminology of e-Oral Health: Consensus Report of the IADR's e-Oral Health Network Terminology Task Force. [Data set]. Zenodo. 10.5281/zenodo.10605383.

## Abbreviations

AI: Artificial intelligence
CNN: Convolutional neural network
EHR: e-Health records
e-OHN: e-Oral health network
IADR: International association of dental research
ML: Machine learning
WHO: World health organization
